# Targeting Th9 cells in autoimmune diseases: a narrative review

**DOI:** 10.3389/fimmu.2025.1615611

**Published:** 2025-07-23

**Authors:** Wang-Dong Xu, You-Yue Chen, Yun-Wei Li, Jing Yang, An-Fang Huang

**Affiliations:** ^1^ Department of Evidence-Based Medicine, Southwest Medical University, Luzhou, Sichuan, China; ^2^ Public Experimental Technology Center, Southwest Medical University, Luzhou, Sichuan, China; ^3^ Department of Rheumatology and Immunology, Affiliated Hospital, Southwest Medical University, Luzhou, Sichuan, China

**Keywords:** Th9 cells, IL-9, autoimmunity, therapeutic target, inflammation

## Abstract

T helper 9 (Th9) cells are a newly identified subset of effector T cells, characterized by their production of IL-9, a hallmark cytokine. Transcription factors such as PU.1 and IRF4 bind to the *IL9* gene promoter and transactivate its expression. IL-9 and its associated transcription factors regulate various aspects of Th9 cell biology, including proliferation, apoptosis, differentiation, and interactions with other immune cells through downstream signaling pathways. In recent years, the involvement of Th9 cells in autoimmune diseases has been widely investigated. Multiple studies have reported the aberrant expression of IL-9, PU.1, and IRF4 in these inflammatory conditions, and functional analyses have demonstrated their significant roles in disease development. In this review, we comprehensively summarize the relationship among Th9-related cytokines, transcription factors, and 14 autoimmune diseases based on both *in vivo* and *in vitro* evidence. We further discuss the regulatory effects of intracellular and extracellular signaling pathways on Th9 cell functions. This compilation of findings may facilitate future research and the development of clinical strategies targeting Th9 cells in autoimmune diseases.

## 1 Introduction

T helper 9 (Th9) cells are a newly identified subset of CD4^+^ effector Th cells, first described in 2008 (1, 2). These cells are primarily induced by the stimulation of CD4^+^ T cells with IL-4 and TGF-β. The primary function of Th9 cells is characterized by their production of IL-9, which is transcribed under the regulation of transcription factors such as PU.1 and IRF4. PU.1 and IRF4 bind to the promoter regions of the *IL9* gene and regulate its expression. IL-9 is recognized as a multifunctional cytokine that exerts effects on various immune and non-immune cell types as well as across different tissues and organs. Its dominant role is to promote the growth and survival of Th9 cells. Additionally, IL-9 has been shown to facilitate the differentiation and proliferation of other Th cells, and Th9 cells are also involved in interactions with other immune cells, including mast cells and macrophages.

Autoimmune diseases are a group of chronic inflammatory conditions that significantly affect the human population worldwide. These diseases are complex and often characterized by unclear pathogenesis, making effective treatment challenging. The body’s immune defense is composed of innate and adaptive immunity. The innate immune system includes monocytes, macrophages, and dendritic cells, whereas the adaptive immune system primarily comprises B and T cells. The immune system maintains physiological homeostasis by either directly clearing pathogens through innate immune cells or by presenting antigens to T and B cells, leading to their activation. When immune cells fail to recognize and eliminate self-antigens in a timely manner, excessive production of autoantibodies may occur, resulting in damage to multiple organs and systems. In this context, various autoimmune diseases—such as systemic lupus erythematosus (SLE) and rheumatoid arthritis (RA)—may be induced—for instance, the serum levels of pro-inflammatory cytokine IL-21 are elevated in patients with SLE, correlating with disease activity. Inhibition of IL-21 signaling has been shown to suppress autoantibody production and reduce disease severity in SLE mouse models (3, 4). Moreover, T follicular helper (Tfh) cells promote B cell differentiation and autoantibody production, and the transfer of Tfh cells into collagen-induced arthritic mice exacerbates disease severity (5). Therefore, autoimmune diseases are often accompanied by abnormal immunological changes. A clear understanding of the pathogenesis of these conditions is essential to design targeted therapies. In recent years, Th9 cells, along with the related inflammatory cytokine IL-9 and its associated transcription factors, have been extensively studied in various autoimmune diseases. Notably, 14 autoimmune diseases—including SLE, RA, antineutrophil cytoplasmic antibody (ANCA)-associated vasculitis, Sjogren’s syndrome (SS), systemic sclerosis (SSc), type 1 diabetes (T1D), Behçet disease (BD), multiple sclerosis (MS), psoriasis, autoimmune thyroid diseases (AITDs), myasthenia gravis (MG), giant cell arteritis (GCA), Takayasu’s arteritis (TAK), and inflammatory bowel disease (IBD)—have demonstrated associations with IL-9. These studies have examined IL-9 expression profiles or IL9 polymorphisms in the respective conditions (**Table 1**) as well as the direct or indirect roles of IL-9, Th9 cells, and their related transcription factors in these associations. In this review, we comprehensively summarize these associations. Furthermore, we focus on how Th9 cell functions are regulated by intracellular and extracellular signaling mechanisms.

**Table 1:** Abnormal expression and proportion of Th9 cells, related transcription factors, and cytokines in autoimmune diseases.

Disease	Sample	Characteristic	Expression/proportion profile	Reference
Systemic lupus erythematosus	PBMC	CD4^+^IL-9^+^ T cell	Increased	37–38^a^
	Urinary	IL-9	Elevated	36^a^
	CD4^+^ T cell	IL-9	Upregulated	38^a^
	Spleen	CD4^+^IL-9^+^ T cell	Increased	39^b^
	Kidney	IL-9^+^ T cell	Elevated	39^b^
	Serum	IL-9	Upregulated	39^b^
Rheumatoid arthritis	PBMC	PU.1^+^, IRF4^+^, CD4^+^IL-9^+^ T cell	Increased	43–46^a^
	Synovial fluid	CD4^+^IL-9^+^ T cell; IL-9, IL-9R, PU.1, IL-4, TGF-β, TSLP	Elevated	30^a^, 43^a^, 46–48^a^
	Plasma	IL-9	Upregulated	43^a^, 47–48^a^
	CD3^+^ T cell	IL-9, IL-9R	Increased	48^a^
ANCA-associated vasculitis	PBMC	CD4^+^IL-9^+^ T cell	Elevated	51^a^
Sjogren’s syndrome	PBMC	CD4^+^IL-9^+^ T cell	Upregulated	52^a^
	Serum	IL-9	Increased	53^a^
	PBMC	IL-9	Increased	52^a^
Systemic sclerosis	PBMC	CD4^+^IL-9^+^ T cell	Increased	55-56^a^
	Plasma	IL-9	Increased	16^a^
	CD4^+^ T cell	IL-9	Increased	16^a^
	Skin	IL-9, IL-9R, IL-4, TSLP, TGF-β	Elevated	55–56^a^
	PBMC	IL-9, IL-9R	Upregulated	55–56^a^
	Serum	IL-9	Increased	55–56^a^
Type 1 diabetes	PBMC	CD4^+^IL-9^+^ T cell	Elevated	57^a^
	Plasma	IL-9	Increased	57^a^
Behçet disease	PBMC, bronchoalveolar lavage	CD4^+^IL-9^+^ T cell	Elevated	58^a^
	PBMC	IL-9, PU.1	Upregulated	58^a^
	Serum, bronchoalveolar lavage	IL-9	Increased	58^a^
Multiple sclerosis	Spinal cord	IL-9	Elevated	60^b^
	Spleen mast cell, astrocyte, oligodendrocyte progenitor cell, oligodendrocyte, microglia	IL-9R	Elevated	61–62^b^
Psoriasis	PBMC	CD4^+^IL-9^+^ T cell	Elevated	69^a^
	Serum	IL-9	Elevated	67^a^
	Dermis	IL-9R	Elevated	68^a^
	Gut	IL-9, IL-9R, PU.1, IRF4	Elevated	69^a^
	Synovium	IL-9, IL-9R	Elevated	69^a^
	Skin	IL-9, IL-9R	Elevated	68^b^
Autoimmune thyroid diseases	PBMC	CD4^+^IL-9^+^ T cell	Elevated	70^a^
	PBMC	IL-9, IRF4	Elevated	70–71^a^
	Plasma	IL-9	Increased	70–71^a^
	Spleen, thyroid tissue	CD4^+^IL-9^+^ T cell	Elevated	72^b^
	Serum	IL-9	Upregulated	72^b^
	Spleen	IL-9, PU.1, IRF-4	Increased	72^b^
Myasthenia gravis	Spleen	CD4^+^IL-9^+^ T cell; PU.1	Elevated	73^b^
Giant cell arteritis	Artery	CD3^+^IL-9^+^, CD4^+^IL-9^+^ T cell; IL-9, TSLP, TGF-β, IL-4, IL-9R	Increased	75–76^a^
Takayasu’s arteritis	PBMC	CD4^+^IL-9^+^, CD4^+^PU.1^+^, IL-9^+^PU.1^+^ T cell	Elevated	77^a^
	Serum	IL-9	Upregulated	77^a^
Inflammatory bowel disease	Lamina propria, epithelium	PU.1^+^, IL-9R^+^, CD3^+^IL-9^+^, CD4^+^IL-9^+^, IRF4^+^IL-9^+^ T cell	Increased	79-83^a^
	Colonic mucosa	IL-9, IRF4, IL-4, TGF-β, PU.1	Elevated	78–80^a^
	Colonic mucosa	CD4^+^IL-9^+^ T cell	Upregulated	84^b^

PU.1, purine-rich nucleic acid binding protein 1; IRF4, interferon-regulatory factor 4; TGF-β, transforming growth factor-beta; TSLP, thymic stromal lymphopoietin; PBMC, peripheral blood mononuclear cell.

a Human.

b Animal.

## 2 Regulation of Th9 cells

**Figure 1:**
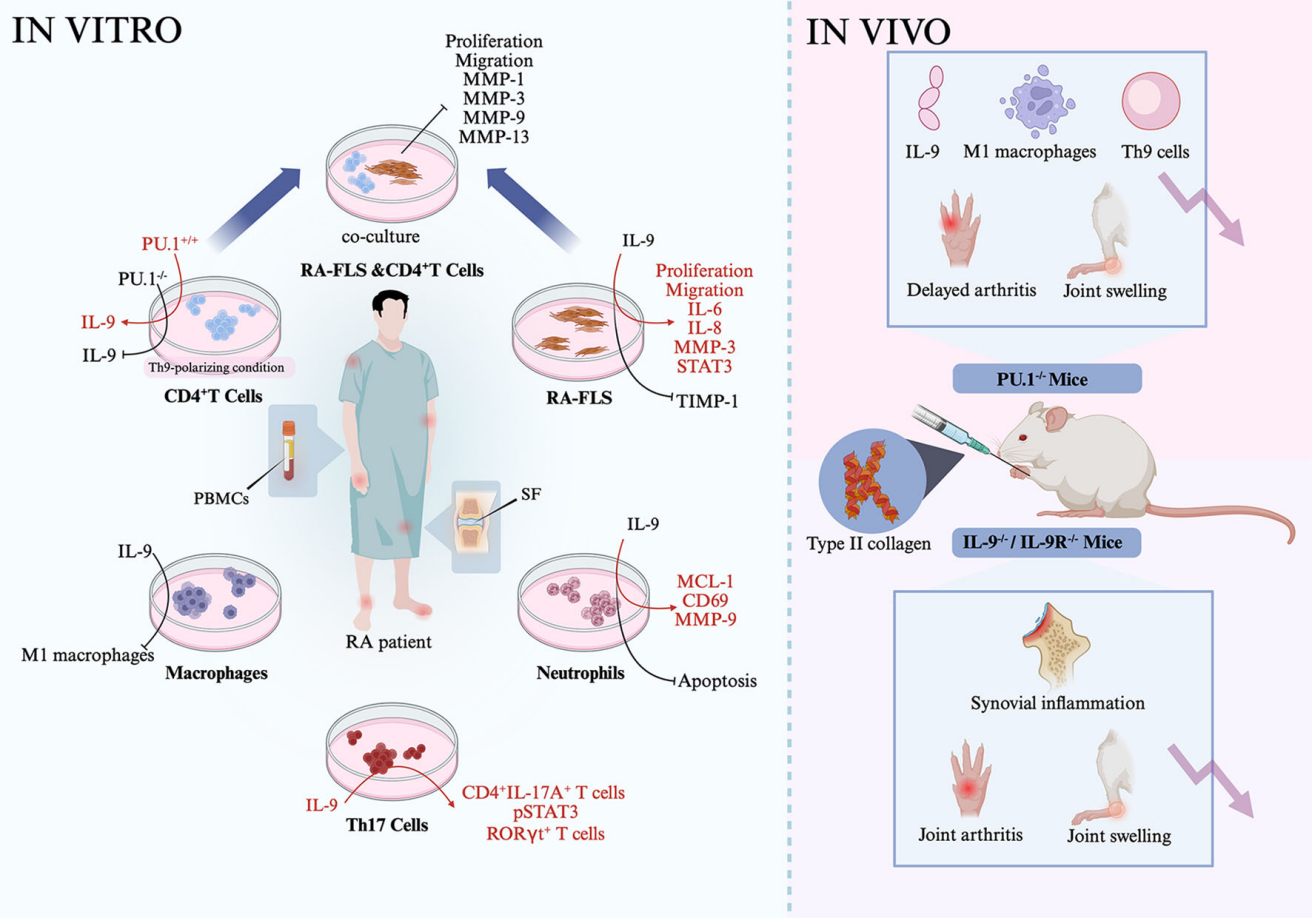
Th9-related signaling. Th9 cell differentiation and function of cytokines, transcription factors such as IL-9 and PU.1 were differently regulated by extracellular signaling, transmembrane proteins, intracellular transcription factors, metabolic components, and pathogen—for instance, inflammatory cytokines IL-1β, TGF-β, IL-4, and IL-2 activate STAT5, which then inhibit BCL-6 activation, leading to the reduced expression of IL-9. ITK interacts with IL-2 and then promotes STAT5 activation, resulting in the downregulated expression of BCL-6 and less IL-9 generation. TL1A activates the STAT6-NF-κB-BATF3 axis and then promotes Th9 differentiation. IFN-γ induces DC generation of IL-27, which then activates STAT1, but inactivates STAT3, leading to Th9 cell proliferation and migration.

Th9 cells, along with their associated transcription factors and cytokines, play critical roles in immune responses. Targeting the functions of Th9 cells may enhance our understanding of their roles in immunity. Furthermore, elucidating the mechanisms that regulate Th9 cells will be valuable to modulate their effects on inflammatory responses (**Figure 1**).

### 2.1 Role of extracellular signaling in Th9 cells

TL1A, a member of the tumor necrosis factor (TNF) superfamily, has been implicated in Th9 cell regulation. Naïve CD4^+^ T cells from wild-type (WT) mice cultured under Th9-polarizing conditions in the presence of TL1A exhibited an elevated expression of IL-9, IL-10, IL-13, and BATF3, along with increased proportions of IL-9^+^IL-10^+^, CD4^+^IL-9^+^, and BATF3^+^IL-9^+^ T cells (6). In contrast, CD4^+^ T cells from BATF3^-/-^ mice showed a reduced expression of IL-9, IL-10, and IL-13 under Th9-polarizing conditions. Moreover, the proportion of BATF3^+^IRF4^+^ cells was reduced in NF-κB p50^-/-^ and STAT6^-/-^ Th9 cells, suggesting that TL1A may promote BATF3 expression and Th9 differentiation via the STAT6- and NF-κB-dependent pathways (6). These effects of TL1A were similar to those of another TNF family member, Fas. When WT naïve CD4^+^ T cells were differentiated under Th9-polarizing conditions, they exhibited increased IL-9 production and higher expression levels of IL-9, PU.1, and IRF4 and several phosphorylated signaling molecules, including NF-κB p65 (pNF-κB p65), p38 (pp38), IKKα (pIKKα), IKKβ (pIKKβ), and IκBα (pIκBα), compared to Fas^lpr^ CD4^+^ T cells (7). When Fas^lpr^ CD4^+^ T cells were differentiated under Th9-polarizing conditions, the addition of a pIκBα or IKK2 inhibitor further suppressed Th9 differentiation. Similarly, PKCβ inhibition inhibited NF-κB p65 activation and reduced the proportion of CD4^+^IL-9^+^ T cells differentiated from Fas^lpr^ CD4^+^ T cells. Similarly, inhibition of Ca^2+^ release in Fas^lpr^ CD4^+^ T cells downregulated the expression of pNF-κB p65, and the addition of a PLC inhibitor to these cells suppressed Ca^2+^ flux. Notably, the treatment of Fas^lpr^ CD4^+^ T cells with a p38 inhibitor suppressed Th9 cell differentiation under Th9-polarizing conditions (7). These findings suggest that Fas promotes Th9 differentiation by positively regulating the PLCγ1–Ca^2+^–PKCβ–NF-κB axis or the p38 pathway. Furthermore, differentiated WT Th9 cells stimulated under IL-2-limiting conditions significantly downregulated the expression of IL-9, BATF3, SGK1, and pNF-κB p65 and upregulated the expression of CSF1 and BCL-6 (8). When IL-2-limited Th9 cells were further stimulated with IL-1β, the proportion of Th9 cells increased, whereas BCL-6 expression was downregulated. Conversely, the addition of an NF-κB inhibitor blocked IL-1β-induced IL-9 production. In BCL-6^+/+^ Th9 cells, IL-9 expression was low, suggesting that IL-1β promotes Th9 cell differentiation by positively regulating the IL-2–NF-κB axis and negatively regulating BCL-6 (8). In addition, ovalbumin-injected mice transfected with an IFN-γ-overexpression vector showed a reduced proportion of CD4^+^IL-9^+^ T cells and CD11c^+^ DCs, downregulated IL-9 expression, and upregulated IL-27 and IL-27R expression in peripheral blood and bronchoalveolar lavage fluid (BALF) (9). CD11c^+^ DCs stimulated with IFN-γ upregulated IL-27 expression, whereas naïve CD4^+^ T cells stimulated with IFN-γ under Th9-polarizing conditions displayed a low expression of IL-9 and pSTAT3, a lower frequency of Th9 cells, and an elevated expression of pSTAT1. When differentiated Th9 cells were cocultured with IFN-γ-stimulated CD11c^+^ DCs, IL-27 expression was increased, while IL-9 expression was decreased (9). Therefore, IFN-γ promotes DC-induced IL-27 production and subsequently inhibits Th9 differentiation through the regulation of the STAT1/3 signaling pathway. Collectively, TL1A, Fas, and IL-1β positively promote Th9 differentiation, whereas IFN-γ signaling inhibits this process.

### 2.2 Effects of transmembrane proteins on Th9 cell differentiation

Transmembrane proteins comprise a group of molecules capable of activating downstream signaling pathways and regulating Th9 cell functions. TLR2, a transmembrane protein, recognizes pathogen-associated molecular patterns and endogenous ligands. When WT naïve CD4^+^ T cells were differentiated into Th9 cells, increased IL-9 expression and a higher proportion of CD4^+^IL-9^+^ T cells were observed. The addition of a TLR2 agonist to Th9 cells significantly increased the proportion of CD4^+^IL-9^+^ T cells and upregulated the expression of IL-9, BATF3, and PU.1 (10). Piezo1 is another transmembrane protein involved in Th9 cell regulation. The overexpression of Piezo1 in WT CD4^+^ T cells led to an increased proportion of CD4^+^IL-9^+^ T cells and increased IL-9 expression under Th9-polarizing conditions (11). Conversely, naïve CD4^+^ T cells from Piezo1^-/-^ mice cultured under Th9-polarizing conditions exhibited a decreased expression of IL-9 and HIF1α and an increased expression of SIRT3, OXPHOS, and SDHA. However, in naïve CD4^+^ T cells from Piezo1^-/-^SIRT3^-/-^ mice under Th9-polarizing conditions, the expression of IL-9 and HIF1α was restored. Similarly, in naïve CD4^+^ T cells from HIF1α^-/-^ mice under Th9-polarizing conditions, an increased proportion of CD4^+^IL-9^+^ T cells and a reduced expression of SIRT3, but an elevated expression of HIF1α, were observed in the presence of an activator of Piezo1. Furthermore, transfection of SDH siRNA into CD4^+^ T cells from Piezo1^-/-^ mice resulted in a higher proportion of CD4^+^IL-9^+^ T cells under Th9-polarizing conditions, suggesting that Piezo1 positively regulates Th9 differentiation through the modulation of the SIRT3–SDHA–OXPHOS–HIF1α axis (11). Similarly, naïve CD4^+^ T cells from epidermal growth factor receptor (EGFR)^-/-^, NOS2^-/-^, or HIF1α^-/-^ mice under Th9-polarizing conditions exhibited a reduced expression of IL-9, HIF1α, PU.1, IRF1, and BATF3 (12). These findings suggest that both Piezo1 and EGFR influence HIF1α signaling and are involved in Th9 cell differentiation and IL-9 production.

### 2.3 Responses of Th9 cells to intracellular transcription factors

Transcription factors are a class of intracellular proteins that regulate gene transcription. Different transcription factors variably influence IL-9 production and Th9 cell function. Naïve CD4^+^ T cells from healthy donors treated under Th9-polarizing conditions exhibited an increased expression of LAT2, SLC7A8, peroxisome proliferator-activated receptor gamma (PPARγ), TRAF6, TAK1, pNF-κB p65, pIκBα, DBP, pSTAT6, Id1, IL-9, IL-5, and IL-9R, along with enhanced glucose uptake and glycolytic activity. Additionally, PPARγ expression correlated with the expression of SLC7A8, IL-5, and IL-9 (13–19). PPARγ plays a central role in lipid and glucose metabolism as well as immune regulation. Inhibition of PPARγ in Th9 cells led to reduced SLC7A8 and pmTORC1 expression and glucose uptake but increased SLC7A11 expression. Under conditions of cysteine deprivation using an SLC7A11 inhibitor, Th9 cells exhibited increased cell death, higher levels of lipid reactive oxygen species (ROS), and decreased glutathione (GSH) expression (14). Inhibition of mTORC1 or adenosine monophosphate-activated protein kinase (AMPK) in Th9 cells reduced IL-9 expression, whereas inhibition of ACC1 doubled both the proportion of CD4^+^IL-9^+^ T cells and the expression of IL-9 (13, 18). Stimulation of IL-9R^+^ T cells with IL-9 promoted T cell proliferation, particularly in high-glucose conditions, indicating that PPARγ supports Th9 cell function by enhancing glycolysis (18). BCL-6 is a nuclear transcriptional repressor. Naïve CD4^+^ T cells transfected with a BCL-6-overexpression vector under Th9-polarizing conditions showed a reduced IL-9 expression and a lower proportion of CD4^+^IL-9^+^ T cells (20). Conversely, CD4^+^ T cells transfected with BCL-6 siRNA led to an increased expression of IL-9. Differentiated CD4^+^IL-9^+^ T cells stimulated with JAK3 or STAT5 inhibitors exhibited an increased BCL-6 expression and decreased IL-9 production. Similarly, STAT5 siRNA-transfected CD4^+^IL-9^+^ T cells showed reduced IL-9 expression but elevated BCL-6 expression (20). Notably, ITK^-/-^ CD4^+^ T cells under Th9-polarizing conditions exhibited a reduced expression of IL-9, IRF4, PU.1, MAF, and pSTAT5 as well as a decreased proportion of CD4^+^IL-9^+^ T cells (21). This was further confirmed in WT Th9 cells treated with an ITK inhibitor, which also showed a lower proportion of CD4^+^IL-9^+^ T cells. However, the addition of IL-2 to ITK^-/-^ Th9 cells upregulated the expression of IL-9 and IRF4. Similarly, ITK^-/-^ Th9 cells transfected with a STAT5-overexpression vector showed a significantly increased expression of IL-9 and IRF4 (21). The introduction of IRF4 into naïve CD4^+^ T cells under Th9-polarizing conditions resulted in a higher percentage of CD4^+^IL-9^+^ T cells (22). These findings suggest that the STAT5–IRF4 axis plays a positive role in Th9 cell function. TAK1, a member of the mitogen-activated protein kinase kinase kinase (MAP3K) family, has multiple roles in cellular signaling pathways. WT Th9 cells treated with a TAK1 inhibitor showed a decreased IL-9 expression and an increased SIRT1 expression (23). Th9 cells transfected with TAK1 siRNA also exhibited a reduced IL-9 expression and an elevated SIRT1 expression. CD4^+^ T cells from SIRT1^-/-^ mice cultured under Th9-polarizing conditions demonstrated an enhanced glycolytic activity, activation of S6 ribosomal protein and HIF1α, and an increased proportion of Th9 cells. However, transfection of SIRT1^-/-^ CD4^+^ T cells with a SIRT1-overexpression vector significantly suppressed the glycolytic activity and inhibited Th9 cell differentiation. In SIRT1^-/-^mTOR^-/-^ mice, both the proportion of CD4^+^IL-9^+^ T cells and IL-9 expression were decreased. In contrast, these parameters were further increased in SIRT1^-/-^HIF1α^-/-^ CD4^+^ T cells under Th9-polarizing conditions (23). Regarding another role of TAK1, both TRAF6^-/-^ Th9 cells and TAK1 inhibitor-treated Th9 cells showed a reduced activation of NF-κB p65 (16). TRAF6^-/-^ Th9 cells also displayed a decreased expression of IL-9 and DBP and an increased expression of E2F8. Inhibition of TAK1 in Th9 cells downregulated DBP expression but increased E2F8 expression. Inhibition of TAK1 in Th9 cells did not produce IL-9 but showed an elevated EF28 expression (16). Similarly, NF-κB p65^-/-^ CD4^+^ T cells cultured under Th9-polarizing conditions exhibited a low IL-9 expression. Treatment of WT Th9 cells with anti-IL-2 or anti-IL-4 antibodies also resulted in reduced IL-9 expression (15). Conversely, the administration of an anti-IFN-γ antibody significantly increased IL-9 expression in Th9 cells (15). These findings demonstrate that the TRAF6–TAK1–NF-κB signaling axis enhances Th9 cell function (**Figure 1**).

The ETS family transcription factor ETV5 has been identified to be associated with Th9. Naïve CD4^+^ T cells from ETV5^-/-^ mice cultured under Th9-skewing conditions exhibited a lower proportion of CD4^+^IL-9^+^ T cells and a reduced expression of IL-9 (24). Overexpression of ETV5 in ETV5^-/-^ Th9 cells resulted in an increased expression of IL-9 and a decreased expression of IL-4. Similarly, naïve CD4^+^ T cells from ETV5^-/-^PU.1^-/-^ mice cultured under Th9-polarizing conditions showed a reduced IL-9 expression and a lower proportion of CD4^+^IL-9^+^ T cells (24). WT Th9 cells transfected with a PU.1-overexpression vector showed an increased frequency of CD4^+^IL-9^+^ T cells, a reduced IL-4 expression, and an enhanced recruitment of GCN5 to the IL-9 gene promoter (25). In contrast, GCN5^-/-^ Th9 cells exhibited limited GCN5 binding to the IL-9 promoter, along with a decreased IL-9 expression. These findings together indicate that the ETV5–PU.1–GCN5 signaling axis transactivates IL-9. In addition, several other transcription factors are involved in Th9 cell regulation. Activin A (ActA) is a Smad activator. Th9 cells stimulated with ActA exhibited an upregulated expression of IL-9, IL-10, and IL-13 and a significantly higher proportion of Th9 cells compared to controls (26). Id1^-/-^ Th9 cells showed a reduced expression of IL-9, GZMA, and CD8, a lower proportion of Th9 cells, and an elevated expression of TCF3 and TCF4 (17). TCF3 and TCF4 can bind to the IL-9 promoter. WT Th9 cells transfected with TCF3- or TCF4-overexpression vectors showed a reduced IL-9 expression and a lower percentage of Th9 cells (17). Moreover, inhibition of the PI3K/AKT pathway in Th9 cells promoted IL-9 production, upregulated FOXO1, KLF2, IRF4, PU.1, and BATF3, and increased the proportion of Th9 cells. However, further inhibition of FOXO1 reversed these effects of PI3K/AKT pathway inhibition on the production of Th9-associated inflammatory proteins (27). Additionally, naïve CD4^+^ T cells from STAT6^-/-^ mice cultured under Th9-skewing conditions showed a reduced IL-9 expression (19). Collectively, the Smad–Id1–FOXO1–STAT6 signaling axis contributes to IL-9 production and Th9 cell differentiation.

### 2.4 Effects of metabolic components on Th9 cells

The inflammatory Th9 immune response is also regulated by metabolic components. WT Th9 cells exposed to oleic acid exhibited an increased percentage of IL-9^+^ cells, whereas the addition of inhibitors that suppress cholesterol or fatty acid synthesis significantly reduced the percentage of CD4^+^IL-9^+^ T cells (28). During Th9 differentiation of naïve CD4^+^ T cells, the expression of genes involved in fatty acid uptake, such as LRP8 and VLDLR, and genes related to fatty acid biosynthesis, including SCD1 and ACSL3, was elevated. An increased expression of palmitate, IL-9, and BATF3 in CD4^+^IL-9^+^ T cells was also observed (29). Notably, exposure of differentiated Th9 cells to an ACC1 inhibitor resulted in a reduced expression of IL-9, BATF3, IRF4, and PU.1. Th9 cells derived from ACC1^-/-^ mice also showed a decreased IL-9 expression and a lower proportion of CD4^+^IL-9^+^ T cells. In contrast, supplementation with palmitic acid or oleic acid promoted IL-9 expression and increased the percentage of CD4^+^IL-9^+^ T cells (29). Therefore, fatty acid metabolism may influence Th9 cell function by interacting with oleic acid-mediated signaling. Treatment of Th9 cells with a TLR2 agonist upregulated the expression of IL-9, IL-33, and IL1RL1 and increased the frequency of CD4^+^IL-9^+^ T cells. However, the addition of calcitriol inhibited TLR2-induced IL-9 expression and suppressed TLR2 and IL-33 expression (30). Neobavaisoflavone (Neo), a traditional Chinese medicine compound with anti-inflammatory activity, was found to reduce the proportion of CD4^+^IL-9^+^ T cells and downregulate IL-9 and PU.1 expression in Th9 cells (31). Overexpression of PU.1 in CD4^+^ T cells reversed this effect, leading to increased IL-9 expression, suggesting that Neo negatively regulates PU.1 and thereby inhibits Th9 cell differentiation.

### 2.5 Pathogen-induced Th9 polarization

*Candida albicans* is an opportunistic commensal fungus capable of morphologically transitioning from yeast to hyphal filaments in response to various environmental stimuli. CD11c^+^ DCs stimulated with yeast-locked *C. albicans* (YLCA) and subsequently cocultured with Rag^-/-^ OT-II CD4^+^ T cells exhibited an elevated IL-9 expression (32). CD11c^+^ DCs stimulated with either YLCA or hyphal-locked *C. albicans* (HCLA) activated the SYK and extracellular signal-regulated kinase (ERK) signaling pathways and upregulated the expression of TNF-α, IL-6, and IL-33. In contrast, coculturing Dectin-1^-/-^ or CARD9^-/-^ DCs with Rag^-/-^ OT-II CD4^+^ T cells did not induce Th9 differentiation. Furthermore, Dectin-1^-/-^ DCs stimulated with HLCA failed to upregulate IL-33 expression. When IL-33^-/-^ DCs were cocultured with Rag^-/-^ OT-II CD4^+^ T cells in the presence of YLCA or HLCA, Th9 cell differentiation was inhibited. Additionally, CARD9^-/-^ or Dectin-1^-/-^ DCs treated with inhibitors of SYK, PLCγ2, p38, or MEK1/2 significantly downregulated IL-33 expression (32). These findings suggest that *C. albicans* activates the Dectin-1–SYK–PLCγ–CARD9–ERK signaling axis in DCs, leading to IL-33 expression and subsequent induction of Th9 immune response.

## 3 Association of Th9 cells, related cytokines, transcription factors, and autoimmunity

### 3.1 SLE

SLE is a systemic autoimmune disease that causes damage to multiple organs and systems (33–35). Patients with SLE and lupus nephritis who received a combination of prednisolone, azathioprine, and mycophenolate exhibited a lower urinary IL-9 expression compared to healthy control individuals (36). Similarly, treatment-naïve, anti-DNA^+^ patients with SLE showed a higher proportion of Th9 cells than controls (37) (**Table 1**). In peripheral blood mononuclear cells (PBMCs) from patients with SLE, IL-9 expression and the percentage of Th9 cells were elevated, whereas the expression of Bach2 was decreased (38). This finding was confirmed in CD4^+^ T cells from patients with SLE, which also showed increased IL-9 and decreased Bach2 expression. When CD4^+^ T cells from patients with SLE were cultured under Th9-polarizing conditions, Bach2 expression remained low. Overexpression of Bach2 in CD4^+^ T cells under Th9-polarizing conditions led to a reduced expression of PU.1, IRF4, and IL-9, along with a lower percentage of Th9 cells. In contrast, silencing Bach2 expression under the same conditions increased IL-9 expression and the percentage of Th9 cells. Notably, co-overexpression of Bach2 and IRF4 in CD4^+^ T cells promoted IL-9 production and increased the percentage of Th9 cells (38). These findings suggest that IL-9 expression and Th9 cell proportions are elevated in patients with SLE and that the transcription factor IRF4 interacts with Bach2 to induce Th9 cell differentiation. In MRL/lpr lupus mice, both the proportion of CD4^+^IL-9^+^ Th9 cells and serum IL-9 levels were higher than those in control mice (39). The serum IL-9 levels correlated with anti-dsDNA antibody expression. In the kidneys of lupus mice, IL-9^+^ cells were heavily infiltrated, and a higher proportion of PNA^+^ germinal center (GC) cells was observed. The proportion of IL-9^+^ cells correlated with the proportion of PNA^+^ GC cells. Following the injection of anti-IL-9 antibody in MRL/lpr mice, renal injury, inflammatory cell infiltration, urinary protein and anti-dsDNA levels, and IL-6, IL-17, ICAM-1, and VCAM-1 expression were all reduced. Anti-IL-9 treatment in these mice also decreased GC cell formation, inhibited B cell proliferation, and downregulated IgM, IgG, IL-17, and STAT3 expression (39). Additionally, treatment with a STAT3 inhibitor significantly suppressed IL-9-induced B cell proliferation and IgM/IgG production. Collectively, these findings indicate that Th9 cells and the associated cytokine IL-9 play a positive role in the pathogenesis of SLE.

### 3.2 RA

RA is a chronic autoimmune disease (40–42) that affects multiple joints, leading to synovial hyperplasia and excessive cartilage erosion. A higher proportion of PU.1^+^ Th9 cells, IRF4^+^ Th9 cells, and CD4^+^IL-9^+^ Th9 cells was observed in the PBMCs of patients with RA than in those of healthy controls (43–46) (**Table 1**). The percentage of Th9 cells was also increased in the synovial fluid (SF) of patients with RA compared to that in patients with OA, and the proportion of Th9 cells in PBMCs and SF was correlated with DAS28 (ESR) scores (43). Plasma IL-9 levels were elevated in patients with RA compared to those in healthy controls and were associated with DAS28 (ESR) scores, CRP levels, and anti-CCP titers (43, 46, 47). The synovial expression of IL-9, IL-9R, TLR2, PU.1, IL-4, TGF-β, and TSLP was also higher in patients with RA than in patients with OA (30, 46–48). In patients with RA, synovial IL-9 expression was positively correlated with the expression of TLR2, CRP, and RF titers but negatively correlated with 25(OH)D3 levels (30). CD3^+^ T cells in the SF of patients with SF showed a high expression of IL-9 and IL-9R (48). IL-9 was predominantly expressed in synovial fibroblasts and infiltrating mononuclear cells in these patients. The proportions of IL-4^+^, TGF-β^+^, and TSLP^+^ cells were each associated with the proportion of IL-9^+^ cells (46). Collectively, Th9 cells, along with related cytokines and transcription factors, appear to be implicated in RA pathogenesis, suggesting that targeting Th9 cells may offer therapeutic potential for RA.

**Figure 2:**
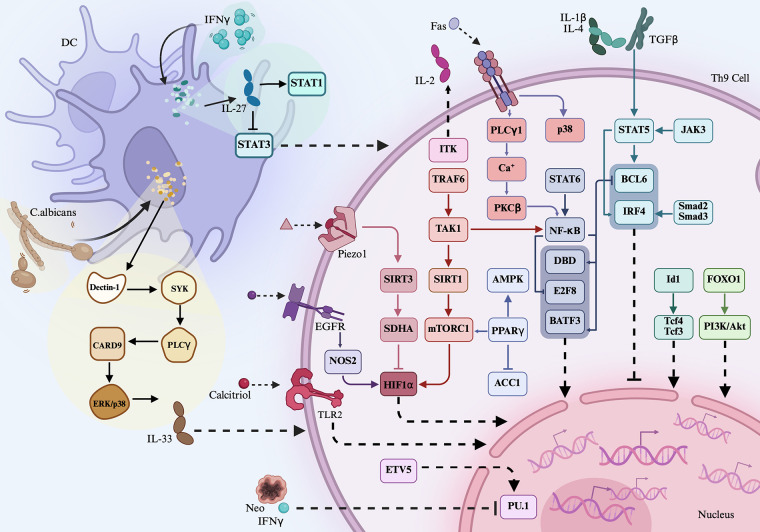
Association of Th9 cells, related cytokines, and transcription factors in rheumatoid arthritis. CD4^+^ T cells, RF-FLS, macrophages, neutrophils, and Th17 cells from RA patients were stimulated with IL-9, showing increased inflammation of the cells. On the contrary, inhibition of PU.1 in the cells abrogated IL-9-mediated effects. Coculturing PU.1^-/-^ Th9 cells with RA-FLS inhibited the proliferation and migration of RA-FLS and downregulated the production of inflammatory matrix metalloproteinases. RA-FLS, rheumatoid arthritis fibroblast-like synoviocytes.

*In vitro* studies have demonstrated the potential involvement of Th9 cell-related transcription factors and cytokines in RA-associated inflammation. When CD4^+^ T cells from patients with RA were cultured under Th9-polarizing conditions, overexpression of PU.1 enhanced IL-9 production, whereas PU.1 inhibition suppressed it (47). Fibroblast-like synoviocytes from patients with RA (RA-FLS) stimulated with IL-9 exhibited increased proliferation and migration. However, FLS proliferation was suppressed when the cells were cultured with an anti-IL-9R antibody (49). Coculturing RA-FLS with conditioned medium from PU.1^-/-^ Th9 cells significantly reduced FLS proliferation and migration and decreased the production of MMP-1, MMP-3, MMP-9, and MMP-13 (47). RA-FLS simulated with TNF-α underwent apoptosis, but this apoptosis was inhibited in the presence of IL-9 (49). Moreover, RA-FLS treated with IL-9 induced an elevated expression of IL-6, IL-8, and MMP-3, reduced the expression of TIMP-1, and promoted the phosphorylation of STAT3. In macrophages from patients with RA, IL-9 stimulation induced M1 macrophage polarization (47). In neutrophils from these patients, IL-9 treatment decreased apoptosis and upregulated the expression of MCL-1, CD69, and MMP-9. Conversely, inhibiting IL-9 expression increased neutrophil apoptosis and reduced the MCL-1 and MMP-9 levels (43). In Th17 cells from patients with RA, IL-9 stimulation of PBMCs and SF samples increased the proportion of CD4^+^IL-17A^+^ and RORγt^+^ T cells, whereas anti-IL-9 antibody treatment reduced the proportion of Th17 cells and inhibited STAT3 phosphorylation (43). In CD3^+^ T cells from patients with RA, IL-9 stimulation enhanced proliferation, which was suppressed by anti-IL-9R antibody treatment (48). Furthermore, stimulation of CD3^+^ T cells with TNF-α induced apoptosis, which was attenuated by IL-9. Notably, IL-9-induced proliferation of CD3^+^ T cells was inhibited in the presence of PI3K/AKT or mTOR pathway inhibitors (48). These findings suggest that the PU.1–IL-9 axis promotes the survival and function of FLS, neutrophils, macrophages, and T cells in patients with RA through downstream signaling pathways (**Figure 2**).

IL-9 has been reported to be involved in the development of RA, and inhibiting IL-9 can reverse disease progression. PU.1^-/-^ mice treated with type II collagen exhibited a delayed onset of arthritis, as evidenced by lower arthritis scores, reduced joint swelling, and milder synovial pathology. These mice also showed a lower IL-9 expression and a decreased proportion of Th9 cells and M1 macrophages (47). In contrast, the injection of exogenous IL-9 significantly accelerated disease progression and increased the percentage of Th9 cells. Similarly, IL-9^-/-^ mice treated with type II collagen demonstrated reduced joint swelling, and IL-9R^-/-^ mice treated with type II collagen showed decreased arthritis severity and fewer swollen joints (47). *Dioscorea nipponica*, a traditional Chinese medicinal herb, contains active compounds such as dioscin (referred to as DDN), which have demonstrated anti-inflammatory effects. In WT mice treated with type II collagen-induced arthritis, treatment with DDN inhibited bone destruction and synovial inflammation. It also downregulated the expression of PU.1, TGF-β, and IRF4 in the synovium, reduced the IL-9 levels in the serum, and decreased the proportion of Th9 cells in the spleen (50). Collectively, these findings suggest that targeting Th9 cells may be an effective strategy for RA treatment.

### 3.3 ANCA-associated vasculitis

ANCA-associated vasculitis (AAV) is a group of autoimmune diseases that primarily affects small blood vessels. Patients with AAV showed an increased percentage of Th9 cells in PBMCs compared to that in healthy controls, and those with active AAV exhibited a significantly higher proportion of Th9 cells than patients with less active disease (51).

### 3.4 SS

SS is characterized by lymphocytic infiltration of the exocrine glands, leading to symptoms such as dry eyes and mouth. A higher proportion of Th9 cells was observed in PBMCs from patients with SS than in healthy control individuals, and this proportion was negatively correlated with the saliva flow rate (52). Serum IL-9 levels were also elevated in SS patients relative to those in controls and were negatively associated with saliva flow rate but positively correlated with serum levels of I-309 in these patients (53). IL-9 expression in PBMCs from patients with SS was higher than that in controls and was associated with globulin expression in these patients (52).

### 3.5 SSc

SSc is an autoimmune disease that primarily affects the skin and lungs, characterized by excessive fibrosis (54). The plasma levels of IL-9 and IL-9 expression in CD4^+^ T cells were elevated in patients with SSc compared to those in healthy controls (16), and high IL-9 expression in CD4^+^ T cells was positively correlated with higher mRSS. An increased expression of IL-9, IL-9R, IL-4, TSLP, and TGF-β was also detected in the skin lesions of patients with SSc. Similarly, IL-9 and IL-9R expression in PBMCs and IL-9 levels in serum were elevated in these patients compared to those in controls (55, 56). The percentage of CD4^+^IL-9^+^ Th9 cells in PBMCs from these patients was also elevated (55, 56). Notably, IL-9 and IL-9R were dominantly expressed in endothelial cells and keratinizing squamous epithelial cells within the skin of SSc patients, and IL-9 was co-localized with PU.1 in these skin lesions. Moreover, neutrophils from these patients stimulated with IL-9 significantly increased IL-8 production and induced NETosis (56). Stimulation of mast cells with IL-9 promoted their proliferation, and B cells stimulated with IL-9 induced Scl70 production. Lung fibroblasts from patients with SSc stimulated with IL-9 differentiated into myofibroblasts (16). Fibroblasts treated with IL-9 showed a high expression of Acta2, Col1a1, MMP-2, and MMP-9. When fibroblasts were cocultured with Th9 cells, the expression of these fibrogenic markers was further elevated. However, treatment with an anti-IL-9 antibody significantly inhibited Th9 cell-induced overexpression of these markers (16). When CD4^+^ T cells were differentiated under Th17-polarizing conditions, a subsequent treatment with an anti-IL-9 antibody inhibited Th17 cell differentiation (55). Additionally, dermal vascular smooth muscle cells (DVSMCs) from patients with SSc stimulated with IL-9 showed an increased expression of IL-17R, collagen I, collagen III, and α-SMA and phosphorylation of p38 and ERK. In contrast, anti-IL-9 antibody treatment suppressed IL-17R expression. DVSMCs stimulated with IL-17 upregulated IL-9R expression, whereas anti-IL-17 antibody treatment suppressed IL-9R expression (55). Furthermore, bleomycin-induced SSc mice treated with an anti-IL-9 antibody exhibited reduced scleroderma, less body weight loss, limited collagen deposition in the skin, and a decreased expression of Acta2, Col1a1, Col1a2, and MMP-2 (16). Collectively, Th9 cells and IL-9 are implicated in the pathogenesis of SSc, and targeting Th9 cell-induced effects may offer therapeutic potential for SSc.

### 3.6 T1D

T1D is a chronic autoimmune disorder caused by the destruction of insulin-producing pancreatic beta cells. Patients with T1D exhibited a higher percentage of CD4^+^IL-9^+^ Th9 cells in PBMCs and elevated plasma levels of IL-9 compared to those in healthy controls (57). Additionally, the percentage of CD4^+^IL-9^+^IL-17A^+^ T cells was increased in these patients. Those with poor metabolic control demonstrated a higher frequency of Th9 cells and plasma IL-9 levels than those in the controls. Plasma IL-9 levels were correlated with HbA1c expression and the percentage of Th9 cells in patients with T1D (57).

### 3.7 BD

BD is a form of vasculitis that manifests with diverse clinical symptoms, including inflammation affecting multiple organs and systems. The frequency of Th9 cells in PBMCs and cells from BALF isolated from patients with BD was higher than those in healthy controls, and patients with active disease exhibited a higher frequency of Th9 cells compared to those with inactive BD (58). Among the patients with active BD, those with pulmonary involvement showed a significantly higher frequency of Th9 cells. The expression of IL-9 and PU.1 in PBMCs from patients with BD was elevated compared to that in controls, with patients with active BD showing higher expression levels than those with less active disease. IL-9 concentrations in both BALF and serum were elevated in patients with BD, particularly in those with pulmonary manifestations. The expression of IL-9 in the serum and BALF was correlated with the frequency of Th9 cells in PBMCs and BALF from patients with BD. Conversely, patients with BD receiving corticosteroid treatment showed a reduced expression of IL-9 and TSLP (58). In contrast, another study found that BD patients had comparable serum levels of IL-9 with healthy controls (59) and that the serum levels of IL-9 in BD patients did not correlate with disease severity. This discrepancy may reflect the influence of treatment on IL-9 expression, suggesting that some patients were in a less active disease state.

### 3.8 MS

MS is an inflammatory disease of the central nervous system (CNS). In autoimmune encephalomyelitis (EAE) mice, the expression of IL-9 and GFAP in the spinal cord was elevated compared to control mice, along with an increased expression of NICD, pSTAT3, IL-6, TNF-α, IP-10, and MCP-1 in both the spinal cord and peripheral blood (60). Similarly, IL-9R was highly expressed in splenic mast cells, astrocytes, oligodendrocyte progenitor cells (OPCs), oligodendrocytes, and microglia of EAE mice (61, 62). Treatment of EAE mice with an anti-IL-9 antibody inhibited disease onset, reduced disease severity, suppressed the CNS expression of CCL5 and VCAM−1, and decreased mast cell infiltration into the CNS (61). Similarly, splenocytes from EAE mice stimulated with an anti-IL-9 antibody showed a reduced proportion of mast cells (61). Astrocytes from EAE mice stimulated with IL-9 exhibited an elevated expression of GFAP, NICD, pSTAT3, IL-6, TNF-α, IP-10, MCP-1, CCL20, CXCL9, and MMP-3 (60, 62). Furthermore, co-stimulation of OPCs with IL-9 and IFN-γ promoted OPC differentiation (62). In contrast, inhibition of Notch1 expression in IL-9-stimulated astrocytes significantly downregulated the expression of pSTAT3, NICD, IL-6, TNF-α, and IP-10 (60). Moreover, EAE mice treated with a mitogen-activated protein kinase (MAPK) signaling inhibitor reduced the proportion of CD4^+^IL-9^+^ T cells in the spleen and inhibited the expression of IL-9 and IRF4 in splenocytes (63). EAE mice treated with a CCR1 antagonist exhibited reduced disease severity and proportions of CD4^+^IL-9^+^, CD3^+^STAT3^+^, and CCR6^+^STAT3^+^ cells in the spleen (64). In addition, NFIL3^-/-^ mice stimulated with MOG_35–55_ displayed a decreased proportion of CD4^+^IL-9^+^ T cells in the spleen and lymph nodes (65). Collectively, Th9 cells and IL-9 directly contribute to EAE pathogenesis and indirectly regulate pro-inflammatory components via MAPK, NFIL3, and CCR1 signaling. However, Th9 cells appear to have a bidirectional effect in MS. Ruocco et al. reported that IL-9 expression in the cerebrospinal fluid (CSF) of patients with relapsing–remitting MS (RR–MS) was negatively correlated with disease severity (66). Stimulation of Th17 cells with IL-9 led to the downregulation of IL-17 and IRF4 expression and activation of the STAT1 and STAT5 signaling pathways. However, IL-9-treated Th17 cells did not inhibit the expression of TNF-α, IL-6, or IFN-γ, nor did they activate STAT3 signaling. These findings suggest that IL-9 may play a protective role in MS (66). The opposing roles of IL-9 in MS may be attributable to several factors. First, the protective effect of IL-9 primarily depends on its ability to suppress Th17 cell function. As previously discussed, the pro-inflammatory effects of IL-9 in MS are mostly associated with the activation of the STAT3 pathway. In contrast, the protective effects may result from the activation of STAT1 and STAT5 signaling without the involvement of STAT3. Additionally, IL-9 stimulation of Th17 cells did not alter the expression of key pro-inflammatory cytokines, such as TNF-α, IL-6, and IFN-γ, which are widely recognized contributors to MS pathogenesis. Therefore, the inability of IL-9 to induce these cytokines may also explain its protective role in MS. Future studies are needed to further investigate this hypothesis.

### 3.9 Psoriasis

Psoriasis is a chronic autoimmune inflammatory skin disorder. Its pathogenesis is associated with immune and genetic predispositions. Serum IL-9 levels were elevated in patients with psoriasis compared to those in healthy controls (67) and were negatively correlated with disease onset age and Nail Psoriasis Severity Index. IL-9R expression was increased in the dermis—particularly at the dermal–epidermal junction—in patients with psoriasis (68). Patients with psoriatic arthritis (PsA) had higher serum levels of IL-9 than those without arthritis (67). Additionally, the expression of IL-9, IL-9R, PU.1, and IRF4 was increased in the gut of patients with PsA, and the percentage of CD4^+^IL-9^+^ T cells was correlated with intestinal inflammation (69). The percentage of CD4^+^IL-9^+^ T cells was also elevated in the peripheral blood of patients with PsA and correlated with disease activity. IL-9 and IL-9R were highly expressed in leukocytic infiltrates and lining layers of PsA synovium (69). Similarly, K5.hTGF-β1 transgenic (psoriasis-prone) mice exhibited a higher expression of IL-9 and IL-9R in the skin compared to control mice (68). Treatment of patients with PsA using anti-TNF or anti-IL-12/IL-23 therapies reduced the proportion of CD4^+^IL-9^+^ Th9 cells. However, stimulation of epithelial cells from these patients with IL-9 upregulated the expression of SOX9 and IL-23p19 (69). Intradermal injection of IL-9 in K5.hTGF-β1 transgenic mice induced epidermal hyperplasia, skin infiltration by CD3^+^ T cells, CD68^+^ monocytes/macrophages, and mast cells, and increased the expression of vascular endothelial growth factor (VEGF) and CD31. Administration of an anti-IL-9 antibody in this model reduced the skin severity scores, inhibited epidermal hyperplasia and dermal inflammatory cell infiltration, and downregulated the expression of IL-17A, STAT3, IFN-γ, VEGF, and CD31 (68). Collectively, both patients with psoriasis and psoriatic animal models show an excessive expression of IL-9 and IL-9R and an altered frequency of Th9 cells, suggesting that IL-9 promotes psoriasis and PsA development.

### 3.10 AITDs

AITDs are a group of chronic autoimmune diseases that primarily affect the thyroid gland, leading to hyperthyroidism and goiter. Patients with AITDs showed a higher expression of IL-9 and IRF4 in PBMCs compared to controls, and both IRF4 and IL-9 expression were associated with elevated IL-17 expression (70). Patients with AITDs also exhibited higher plasma levels of IL-9 than those observed in healthy controls, and these levels were correlated with TgAb expression. Similarly, the percentage of Th9 cells was increased in patients with AITDs and was also associated with TgAb expression (70). Regarding *IL9* gene polymorphisms, the rs2069879 polymorphism was correlated with AITDs, whereas rs31564, rs1859430, and rs2069868 were not associated with AITD genetic susceptibility in the Chinese Han population (70). As a subtype of AITDs, patients with Graves’ disease (GD) had a higher percentage of Th9 cells and an elevated expression of Foxo1, IRF4, IL-9, and IL-17 in PBMCs compared to those in controls (71). In contrast, patients with GD in remission showed a lower expression of IL-9 and IL-17. IRF4 expression was significantly higher in patients with positive TRAb status. Notably, the plasma levels of IL-9 were elevated in patients with GD compared to those in healthy controls and were correlated with TRAb expression and the percentage of Th9 cells (71). Moreover, in mouse models of AITDs, the serum levels of TgAb and IL-9 were higher than those in control mice (72). The proportion of Th9 cells in the spleen and thyroid tissue was elevated, along with an increased expression of IL-9, PU.1, and IRF4 in splenocytes (72). When IL-9 was used to stimulate PBMCs from patients with GD, IFN-γ expression was increased (70). Collectively, Th9 cells, IL-9, and the related transcription factors were highly expressed in AITDs.

### 3.11 MG

MG is one of the most well-characterized autoimmune disorders to date. Experimental autoimmune myasthenia gravis (EAMG) rats exhibited a higher proportion of Th9 cells and an elevated expression of PU.1 in splenocytes compared to control mice (73). Administration of an anti-IL-9 antibody to EAMG rats significantly reduced the clinical scores, mitigated weight loss, delayed disease onset, inhibited Th1 cell differentiation, and promoted Treg cell differentiation (73). On the contrary, injection of recombinant rat IL-9 (rrIL-9) into EAMG rats decreased the clinical scores, dampened weight loss, delayed EAMG development, decreased the proportion of Th1 cells, and increased the proportion of Treg cells (74). These findings suggest that IL-9 may differentially regulate the onset and progression of EAMG, potentially because of exogenous IL-9 competing with endogenous IL-9, thereby antagonizing its effects in this animal model. Notably, both studies demonstrated comparable changes in clinical and laboratory parameters. However, these studies employed different treatment strategies (anti-IL-9 antibody vs. rrIL-9) and used different doses of each agent. Therefore, the therapeutic effects and underlying mechanisms of IL-9 modulation in EAMG may vary.

### 3.12 GCA and TAK

GCA is the most common disorder of large-vessel vasculitis, whereas TAK is a granulomatous vasculitis. Both diseases share a similar pathogenesis, primarily involving autoimmune inflammation of the aorta and its major branches. In patients with GCA, arteries exhibiting classic transmural inflammation revealed an elevated expression of IL-9, TSLP, TGF-β, IL-4, IL-8, and IL-9R in vascular smooth muscle cells (VSMCs), endothelial cells, and neutrophils (75, 76). IL-9 expression was correlated with the expression of ESR, CRP, and IL-17 in patients with GCA. Co-localization of IL-9 with PU.1 and IL-9 with IL-17 was observed in giant cells throughout inflamed arteries (76). Similarly, the inflamed arteries from patients with GCA exhibited increased clusters of CD3^+^IL-9^+^ and CD4^+^IL-9^+^ T cells compared to those in controls (75). Administration of IL-9 in GCA mouse models resulted in aggravated inflammation, as evidenced by severe destruction of arterial wall structures, significant loss of the medial layer, and “motheaten” areas within the residual media and intima. Moreover, inflamed arteries in IL-9-treated GCA mice exhibited a greater proportion of CD3^+^ T cells and a higher expression of IFN-γ, IL-17, IL-21, CD80, CD86, IL-1β, IL-6, and TNF-α (75). In contrast, injection of an anti-IL-9 antibody into GCA mice alleviated inflammation across all arterial wall layers and reduced the secretion of IFN-γ, IL-17, IL-21, IL-1β, IL-6, and TNF-α (75). Furthermore, the serum levels of IL-9 were higher in patients with TAK than in healthy controls, along with an increased percentage of CD4^+^IL-9^+^ and CD4^+^PU.1^+^ T cells in PBMCs (77). The frequency of IL-9^+^PU.1^+^ T cells was also increased in patients with TAK, and the serum IL-9 levels correlated with ESR expression (77). Collectively, these findings indicate that IL-9 is positively associated with the pathogenesis of both GCA and TAK.

### 3.13 IBD

IBD is an autoimmune disorder associated with gut microbiota dysbiosis. In the inflamed colonic mucosa of patients with active IBD, the expression of ETV5 and IL-9 was elevated, and IL-9 expression correlated with the expression of ETV5 (78). An increased expression of IL-9, IRF4, Smad2, Smad3, IL-17A, IL-6, IL-4, IL-10, IL-13, IL-21, IFN-γ, TGF-β, TNF-α, IRF4, and PU.1 was observed in colonic biopsies of patients with ulcerative colitis (UC), and IL-9 expression was related to the expression of IRF4, Smad2, Smad3, IL-6, IL-13, and IL-17A (79, 80). A high expression of IL-9 was associated with disease activity in patients with UC (81). Elevated percentages of PU.1^+^, IL-9R^+^, CD3^+^IL-9^+^, CD4^+^IL-9^+^, and IRF4^+^IL-9^+^ T cells were observed in the lamina propria and epithelium of patients with UC and Crohn’s disease (CD) compared to those in healthy controls (79–83). These findings were confirmed in a dextran sodium sulfate (DSS)-induced colitis mouse model, which showed a higher percentage of CD4^+^IL-9^+^ T cells in the intestinal mucosa compared to the control mice (84). Collectively, these results indicate that Th9 cells are involved in IBD pathogenesis.

With respect to the role of IL-9 and Th9 cells in colitis-associated inflammation, the overexpression of ETV5 in CD4^+^ T cells from patients with IBD under Th9-polarizing conditions significantly promoted Th9 cell differentiation and increased the expression of IL-9 and IRF4 (78). Conversely, CD4^+^ T cells transfected with both ETV5 overexpression and IRF4 knockdown under Th9-polarizing conditions showed a reduced IL-9 expression and a lower percentage of Th9 cells. Stimulation of colonic fibroblasts from patients with IBD with IL-9 led to an elevated expression of Col1a1, Col3a1, collagen I, collagen III, α-SMA, and TAF1. Overexpression of TAF1 in these fibroblasts similarly upregulated Col1a1, Col3a1, collagen I, collagen III, and α-SMA. In contrast, silencing TAF1 expression in IL-9-stimulated fibroblasts reduced the expression of Col1a1, Col3a1, collagen I, and collagen III, suggesting that IL-9 interacts with ETV5 and subsequently regulates TAF1, thereby contributing to the production of colitis-associated inflammatory factors (78). Polymorphonuclear leukocytes from the peripheral blood of patients with UC treated with IL-9 showed an elevated expression of IL-8 and reduced apoptosis (80). This effect was reversed by treatment with an anti-IL9 antibody, which suppressed IL-8 production. IL-9 stimulation of epithelial cells in patients with UC led to an upregulated pSTAT5 expression. In Caco-2 monolayers, a model of intestinal epithelial cells, IL-9 treatment inhibited cell growth, whereas anti-IL-9 antibody promoted growth, indicating that IL-9 blockade may facilitate epithelial wound healing in the gut (80). With respect to the role of IL-9 and Th9 cells in colitis pathogenesis, adoptive transfer of Th9 cells into colitis mice induced weight loss, shortened colon length, increased leukocyte infiltration, and damaged glandular architecture. Conversely, treatment of colitis mice with an anti-IL-9 antibody resulted in the reduced expression of IL-6, TNF-α, IL-1β, IL-10, and IL-22 in colonic tissues and decreased percentage of PU.1^+^ Th9 cells in the lamina propria and improved the clinical outcomes (7, 81, 83). IL-9^-/-^ mice treated with TNBS exhibited attenuated colitis compared to control mice treated with TNBS alone. This was evidenced by reduced mucosal inflammation, less weight loss, a lower percentage of CD4^+^PU.1^+^ cells in the intestinal mucosa, and a decreased expression of the tight junction molecule claudin-1 (85). Notably, claudin-2 expression remained unchanged in IL-9^-/-^ mice following TNBS treatment (85). TNBS-induced colitis mimics the key features of CD in humans, which is distinct from UC, a condition characterized by Th1-type mucosal immune responses. These findings suggest that IL-9 deficiency confers protection against TNBS-induced colitis (85). However, these results contrast with the findings of Gerlach et al., who reported that Th9 cells inhibited disease progression in a UC model (81). In their study, IL-9^-/-^ mice treated with oxazolone showed a reduced claudin-2 expression compared to oxazolone-treated WT mice, while caudin-1 expression remained unchanged (81). Given that claudin-1 and claudin-2 play distinct roles in epithelial barrier integrity, the differential regulation of these proteins by IL-9 across various colitis models may underlie the context-dependent role of IL-9/Th9 cells in colitis development. Additionally, DSS-treated mice with PU.1^-/-^ T cells showed improved colitis and reduced the tumor burden compared to controls (86). Collectively, these findings indicate that Th9 cells regulate colitis pathogenesis.

## 4 Future directions

Targeting the Th9/IL-9 axis is a promising approach for immunotherapy in autoimmune diseases. Although direct clinical trials remain limited, several studies have indirectly explored the potential of modulating the Th9/IL-9 axis in autoimmune contexts—for instance, mononuclear cells from patients with RA treated with sRANKL and M-CSF in the presence of IL-9 showed enhanced osteoclast formation and function as well as an elevated expression of MMPs (87). These findings suggest that IL-9 contributes to osteoclastogenesis and joint structural damage and that targeting it may have therapeutic benefits in RA. In another study, the administration of the traditional Chinese medicine Xiaoyin Jiedu granules to patients with psoriasis vulgaris significantly reduced the disease severity, along with decreased proportions of Th9 cells and a reduced expression of PU.1 and IL-9 (88). Similarly, patients with RR-MS who received IFN-β treatment for 2 months showed a reduction in serum IL-9 levels (89). Moreover, therapeutic strategies directly targeting Th9/IL-9 have been explored in non-autoimmune settings—for instance, intratumoral delivery of IL-9 via an oncolytic vaccinia virus in a colon cancer mouse model demonstrated antitumor effects as evidenced by the increased expression of IFN-γ, granzyme B, and perforin as well as a higher proportion of regulatory T cells (90). Notably, patients with mild asthma who were treated with a humanized anti-IL-9 monoclonal antibody (MEDI-528) reported fewer asthma exacerbations and reduced post-exercise FEV1 decline than patients treated with a placebo (91). Collectively, these findings support the potential of targeting Th9/IL-9 in autoimmune diseases; however, clinical trials are needed to evaluate its efficacy and safety across various autoimmune conditions.

## 5 Conclusion

This review extensively discussed the influence of extracellular signaling pathways, transmembrane proteins, intracellular transcription factors, metabolic components, and pathogens on Th9 cell function and associated cytokine activity in the context of autoimmune diseases. Moreover, the expression patterns of Th9-related transcription factors and cytokines, as well as the distribution of Th9 cells, were summarized based on current research findings. However, several important considerations require further exploration—for instance, emerging studies have shown that Th9-stimulating proteins derived from *Haemonchus contortus* may be promising candidates for vaccine development owing to their immunomodulatory properties. These proteins can induce Th9 immune responses, potentially enhancing protective immunity against helminth infections (92). Therefore, the role of Th9-induced vaccines in the prevention or modulation of autoimmune diseases is worth investigating. Moreover, most current functional studies have been conducted in animal models, whereas data on IL-9 expression, transcription factor profiles, and Th9 cell distribution in human autoimmune diseases are primarily derived from case–control studies. Longitudinal cohort studies are needed to better assess their potential as markers of autoimmune diseases. In summary, Th9 cells play a pivotal role in inflammatory immune responses, offering new insights into autoimmune homeostasis. Targeting Th9 cells may provide a promising avenue for the development of novel therapies to prevent or treat autoimmune diseases.
